# Long Non-Coding RNA *AGAP2-AS1*: A Comprehensive Overview on Its Biological Functions and Clinical Significances in Human Cancers

**DOI:** 10.3390/molecules29153461

**Published:** 2024-07-24

**Authors:** Feng Ma, Bingbing Zhang, Yiqi Wang, Chenghua Lou

**Affiliations:** School of Pharmaceutical Sciences, Zhejiang Chinese Medical University, Hangzhou 310053, China; 202112211303034@zcmu.edu.cn (F.M.); 20041022@zcmu.edu.cn (B.Z.)

**Keywords:** *AGAP2-AS1*, biological function, clinicopathological character, oncogene, mechanism

## Abstract

Long non-coding RNAs (lncRNAs) are well known for their oncogenic or anti-oncogenic roles in cancer development. *AGAP2-AS1*, a new lncRNA, has been extensively demonstrated as an oncogenic lncRNA in various cancers. Abundant experimental results have proved the aberrantly high level of *AGAP2-AS1* in a great number of malignancies, such as glioma, colorectal, lung, ovarian, prostate, breast, cholangiocarcinoma, bladder, colon and pancreatic cancers. Importantly, the biological functions of *AGAP2-AS1* have been extensively demonstrated. It could promote the proliferation, migration and invasion of cancer cells. Simultaneously, the clinical significances of *AGAP2-AS1* were also illustrated. *AGAP2-AS1* was exceptionally overexpressed in various cancer tissues. Clinical studies disclosed that the abnormal overexpression of *AGAP2-AS1* was tightly connected with overall survival (OS), lymph nodes metastasis (LNM), clinical stage, tumor infiltration, high histological grade (HG), serous subtype and PFI times. However, to date, the biological actions and clinical significances of *AGAP2-AS1* have not been systematically reviewed in human cancers. In the present review, the authors overviewed the biological actions, potential mechanisms and clinical features of *AGAP2-AS1* according to the previous studies. In summary, *AGAP2-AS1*, as a vital oncogenic gene, is a promising biomarker and potential target for carcinoma prognosis and therapy.

## 1. Introduction

Nowadays, carcinoma is a leading cause of fatality in a great number of countries worldwide. The cancer occurrences and mortalities were greatly increasing year by year [1,2]. In 2020, there were around 19.3 million novel cases and 10.0 million deaths in the world [1]. Notably, breast, lung, colorectal, prostate and stomach cancers were the most commonly diagnosed carcinomas. Although great efforts have been made to investigate the possible cancerogenic mechanisms and develop novel anticancer agents, the number of deaths are still increasing.

LncRNAs are specific RNA molecules longer than 200 nucleotides, which do not encode proteins [3]. Theoretically, intergenic region, 5′/3′-untranslational regions, intron and exon could transcribe lncRNAs. Then, the intricate second structures were formed, which further interacted with RNA, DNA and proteins [4]. The mechanisms of lncRNAs regulating gene expression were quite complicated, including preventing the degradation of mRNA, modulating transcription factors, binding to promoters to regulate targeted gene expression and regulating macromolecule–protein interactions [5,6]. Momentously, the biological activities of lncRNAs were extensively verified, such as being involved in cell differentiation, cell cycle regulation and epigenetic control. Functionally, abundant evidence has confirmed that lncRNAs could display both oncogenic or anti-oncogenic roles in cancers. They could promote tumorigenesis at different stages via interacting with cancer-associated signaling pathways [7,8]. Notably, some lncRNAs were even verified as hallmarks in human cancers [9,10,11].

**Figure 1:**
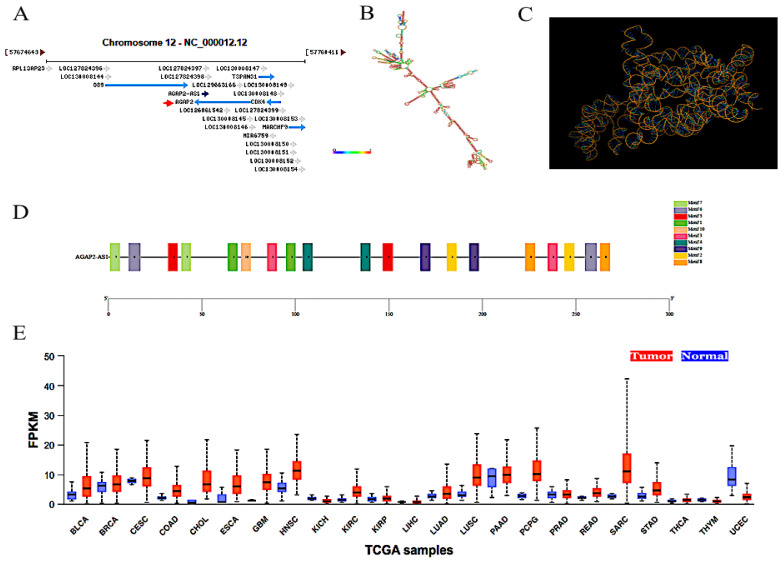
Related information of *AGAP2-AS1*. (**A**) The genomic localization of *AGAP2-AS1* (https://www.ncbi.nlm.nih.gov, accessed on 6 July 2024). (**B**) Secondary structure of *AGAP2-AS1*. (**C**) Three-dimensional structure of *AGAP2-AS1*. (**D**) Motif analysis of *AGAP2-AS1*. (**E**) The expression level of *AGAP2-AS1* in clinical carcinomatous (red color) and non-carcinomatous (blue color) tissues was analyzed using the UALCAN database (https://ualcan.path.uab.edu/, accessed on 18 May 2024). BLCA: Bladder urothelial carcinoma; BRCA: Breast invasive carcinoma; CESC: Cervical squamous cell carcinoma; CHOL: Cholangiocarcinoma; COAD: Colon adenocarcinoma; ESCA: Esophageal carcinoma; GBM: Glioblastoma multiforme; HNSC: Head and neck squamous cell carcinoma; KICH: Kidney chromophobe; KIRC: Kidney renal clear cell carcinoma; KIRP: Kidney renal papillary cell carcinoma; LIHC: Liver hepatocellular carcinoma; LUAD: Lung adenocarcinoma; LUSC: Lung squamous cell carcinoma; LIHC: Liver hepatocellular carcinoma; LUAD: Lung adenocarcinoma; LUSC: Lung squamous cell carcinoma; PAAD: Pancreatic adenocarcinoma; PCPG: Pheochromocytoma and paraganglioma; PRAD: Prostate adenocarcinoma; READ: Rectum adenocarcinoma; SARC: Sarcoma; THYM: Thymoma; THCA: Thyroid carcinoma; UCEC: Uterine corpus endometrial carcinoma.

*AGAP2-AS1*, a novel lncRNA (1567 nt in length), is transcribed by a gene situated in 12q14.1 (Figure 1A) [12]. The secondary and three-dimensional structures of *AGAP2-AS1* are illustrated in Figure 1B,C. Motif analysis indicated that 10 different motifs were identified in *AGAP2-AS1*, which were sequentially named as motifs 1–10 (Figure 1D). As expected, the motifs were conserved in composition. Moreover, motif 1, motif 4, motif 7 and motif 8 were found to be the core motifs. Notably, all core motifs appeared twice. Currently, studies regarding the biological functions of *AGAP2-AS1* are mainly focusing on cancer, psoriasis pathogenesis, pre-eclampsia and systemic sclerosis [12,13,14,15]. Interestingly, most of the studies illustrated the biological roles of *AGAP2-AS1* in cancers. As an antisense lncRNA transcribed from *AGAP2*, *AGAP2-AS1* was widely involved in the progression of cancers [12,16]. According to the reports, *AGAP2-AS1* was demonstrated to be abnormally overexpressed in cancers [12]. The *AGAP2-AS1* level in tumors was much higher than that in normal tissues (Figure 1E). It was conventionally correlated with poor OS and DFS [17]. Importantly, the oncogenic functions of *AGAP2-AS1* were also extensively documented, including promotion of proliferation, migration, invasion and tumorigenesis [18,19,20]. However, to date, there have been no systematical and comprehensive reviews regarding *AGAP2-AS1* in human cancers. Therefore, the aim of the present overview was to summarize the biological roles and potential molecular mechanisms of *AGAP2-AS1* according to the previous studies. Meanwhile, the clinical significances of *AGAP2-AS1* were also illustrated. In summary, as a vital oncogene, *AGAP2-AS1* is a promising biomarker and potential target for cancer therapy.

## 2. Biological Functions

**Figure 2:**
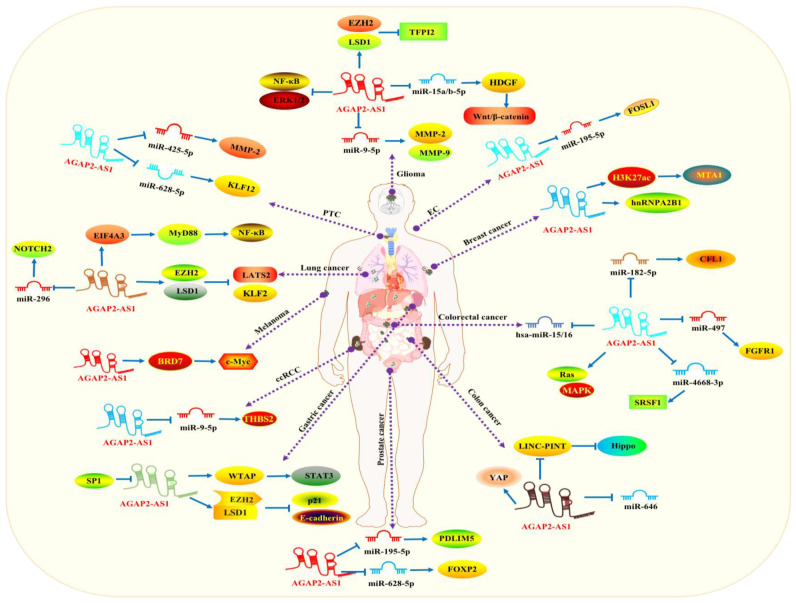
The potential molecular mechanisms of *AGAP2-AS1* in human carcinomas. *AGAP2-AS1* promoted cancer proliferation, migration and invasion in various cancers, including glioma, PTC, LC, melanoma, ccRCC, GC, PC, EC, BC, CRC and CLC.

A large quantity of studies have testified to the aberrant expression of *AGAP2-AS1* in human malignancies, including glioma, colorectal, lung, ovarian, prostate, breast, cholangiocarcinoma, bladder, colon and pancreatic cancers. Results indicated that *AGAP2-AS1* could promote cancer progression mainly via facilitating cancer proliferation, migration and invasion. Meanwhile, the related molecular mechanisms were also documented (Figure 2 and Table 1).

### 2.1. Glioma

Glioma, the most universally arising neuroepithelial cancer, principally occurs in the brain and arises in the glia [21,22]. Importantly, the biological actions of *AGAP2-AS1* in neuroglioma were demonstrated in some studies [23,24]. Experimental results illustrated that the aberrant expression of *AGAP2-AS1* was tightly linked with cancer invasion, multiplication and migration. Wang et al. verified that high expression of *AGAP2-AS1* was notably detected in LN229 and U87MG cells. Inhibition of *AGAP2-AS1* remarkably decreased the proliferation, migration and invasion of cancer cells. Meanwhile, Annexin-V staining showed that the number of apoptotic cells were obviously increasing [23]. Similar results were also demonstrated in some other cell lines, such as U87, U251, A172, LN229 and SHG44 cells [19,25,26]. Importantly, the potential mechanisms were also reported. Luo et al. illustrated that *AGAP2-AS1* interacted with EZH2 and LSD1, and recruited them to the TFPI2 promoter region to restrain its transcription, thereby exerting oncogenic functions [25]. Moreover, Luo et al. constructed a ceRNA network of *AGAP2-AS1*-miR-9-5p-MMP-2/MMP-9. Enrichment analysis predicted that *AGAP2-AS1* could facilitate the migration, proliferation and differentiation of glioma cells via sponging miR-9-5p [27]. In U87, U251 and LN229 cells, *AGAP2-AS1* could advance cancer proliferation via sponging miR-15a/b-5p to increase the HDGF level [26]. Furthermore, suppression of *AGAP2-AS1* obviously restrained the progression of cancer cells via downregulating the levels of NF-κB and Erk1/2 [28]. The above results indicated that *AGAP2-AS1* promoted the proliferation, migration and invasion of glioma cells mainly through regulating miR-15a/b-5p/HDGF/Wnt/β-catenin, EZH2/LSD1/TFPI2, miR-9-5p-MMP-2/MMP-9 and NF-κB/Erk1/2 pathways.

**Table 1:** The biological functions and related molecular mechanisms of *AGAP2-AS1* in human carcinomas.

Cell Lines	Expression Status	Role	Function	Regulatory Mechanism	Ref.
Glioma					
U87 and U251 cells	Upregulation	Oncogene	↑ Proliferation ↑ Metastasis ↑ Invasion ↓ Apoptosis	/	[19]
LN229 and U87MG cells	Upregulation	Oncogene	↑ Proliferation ↑ Metastasis ↑ Invasion ↓ Apoptosis	/	[23]
U87, U251 and LN229 cell lines	Upregulation	Oncogene	↑ Proliferation ↓ Apoptosis	miR-15a/b-5p/HDGF/Wnt/β-catenin axis	[26]
U87/MG and U251/MG cells	Upregulation	Oncogene	↑ Proliferation ↑ Invasion ↓ Apoptosis ↑ Tumor growth	EZH2/LSD1/TFPI2	[25]
/	Upregulation	Oncogene	↑ Proliferation ↑ Metastasis ↑ Invasion	miR-9-5p/MMP-2/MMP-9 axis	[27]
T98G, U251 and LN229 cells	Upregulation	Oncogene	↑ Proliferation ↑ Migration ↑ Invasion	NF-κB/Erk1/2	[28]
LATS2 and KLF2 cells	Upregulation	Oncogene	↑ Proliferation ↑ Metastasis ↑ Drug resistance ↓ Apoptosis	NOTCH, ERBB, RIG, NOD and JAK/STATA pathways	[29]
LC					
A549, ltp-2, SPCA1, H157 and NIH-H358 cell lines	Upregulation	Oncogene	↑ Proliferation ↑ Migration ↑ Drug resistance	miR-296/NOTCH2	[30]
MRC-5 cells	Upregulation	Oncogene	↑ Proliferation ↑ Metastasis	EIF4A3/MyD88/NF-κB pathway	[31]
H1299 and H1975 cells	Upregulation	Oncogene	↑ Proliferation ↑ Invasion ↑ Migration ↓ Apoptosis	LATS2/KLF2/EZH2/ LSD1	[32]
CRC					
LoVo and SW480 cells	Upregulation	Oncogene	↑ Growth ↑ Migration ↑ Invasion ↑ EMT	E2F4/miR-182-5p/CFL1 axis	[20]
DLD-1 and RKO cells	Upregulation	Oncogene	↑ Proliferation ↓ Apoptosis ↑ Migration ↑ Invasion	miR-497/FGFR1 axis	[33]
SW480 and HT29 cells	Upregulation	Oncogene	↑ Proliferation ↑ Migration	hsa-miR-15/16 family	[34]
DLD-1 and HT29 cells	Upregulation	Oncogene	↑ Proliferation	Ras/MAPK pathway	[35]
SW620 and HT-29 cells	Upregulation	Oncogene	↑ Proliferation ↑ Migration ↑ Invasion ↑ EMT	miR-4668-3p/SRSF1 axis	[36]
CLC					
RKO and HCT116 cells	Upregulation	Oncogene	↑ Proliferation ↑ Invasion ↑ Migration	LINC-PINT/Hippo signaling	[37]
SW480 and HCT-116 cells	Upregulation	Oncogene	↑ Proliferation ↓ Apoptosis	/	[38]
HCT116 cells	Upregulation	Oncogene	↑ Proliferation ↓ Apoptosis	miR-646	[39]
SW480 and HCT-116 cells	Upregulation	Oncogene	↑ Proliferation ↑ Migration ↓ Apoptosis	YAP pathway	[40]
OC					
OVCAR3 and A2780 cells	Upregulation	Oncogene	↑ Proliferation	MEG3	[15]
SKOV3-ip, OVCAR3, HO8910, HEY and ES2 cell lines	Downregulation	Anti-oncogene	↓ Proliferation ↓ Migration ↓ Invasion	KRAS/FGFR4/CTSK/EMT	[16]
PCa					
VCaP, 22Rv1, CRL-1740, CRL-2422 and PC3M cell lines	Upregulation	Oncogene	↑ Proliferation ↑ Migration ↑ Invasion ↑ Tumor growth	miR-195-5p/PDLIM5 axis	[41]
PCa cells	Upregulation	Oncogene	↑ Proliferation ↑ Migration ↑ Invasion ↑ EMT ↓ Apoptosis	miR-628-5p/FOXP2/WNT axis	[42]
BC					
MCF-7, BT-474, SK-BR-3 and MDA-MB-231 cell lines	Upregulation	Oncogene	↑ Proliferation ↓ Apoptosis	HuR/H3K27ac/MTA1	[43]
SKBR-3 and BT474 cells	Upregulation	Oncogene	↑ Cell viability ↑ Trastuzumab resistance	hnRNPA2B1	[12]
CHOL					
RBE and HuCCT1 cells	Upregulation	Oncogene	↑ Proliferation ↓ Apoptosis	EZH2/CDKN1A	[44]
RBE and HuCCT1 cells	Upregulation	Oncogene	↑ Proliferation ↑ Migration ↑ Invasion	/	[45]
BLCA					
UM-UC-3 and T24 cells	Upregulation	Oncogene	↑ Proliferation ↑ Migration	/	[46]
5637 and T24 cells	Upregulation	Oncogene	↑ Proliferation ↑ Migration ↑ Invasion ↑ Angiogenesis ↑ Tumor growth	IGF2BP2/LRG1	[47]
RCC					
786-O and ACHN cells	Upregulation	Oncogene	↑ M2 polarization ↑ Malignant behavior ↑ Proliferation ↑ Tumor growth	miR-9-5p/THBS2/PI3K-AKT axis	[48]
PTC					
K1 and TPC1 cells	Upregulation	Oncogene	↑ Migration ↑ Invasion	miR-425-5p/MMP-2 axis	[49]
BCPAP, KTC-1, NIM1 and TPC1 cell lines	Upregulation	Oncogene	↑ Proliferation ↑ Migration ↑ Invasion	miR-628-5p/KLF12 axis	[50]
GC					
AGS and HGC-27 cells	Upregulation	Oncogene	↑ Proliferation ↑ Migration	WTAP/METTL3/METTL14/STAT3 axis	[51]
BGC-823 and AGS cells	Upregulation	Oncogene	↑ Proliferation ↑ Migration ↑ Invasion ↑ Tumor growth	LSD1/EZH2/CDKN1A/E-cadherin transcription	[52]
EC					
KYSE70, KYSE-510 and EC9706 cell lines	Upregulation	Oncogene	↑ Proliferation ↓ Apoptosis ↑ Migration ↑ Invasion ↑ Tumorigenesis	miR-195-5p/FOSL1 axis	[53]
Melanoma					
A375 and A875 cells	Upregulation	Oncogene	↑ Proliferation ↑ Colony formation ↑ Migration	BRD7/c-Myc pathway	[54]
LSCC					
AMC-HN-8 and Tu-177 cells	Upregulation	Oncogene	↑ Proliferation ↑ Invasion	miR-193a-3p/LOXL4 axis	[55]
PC					
AsPC-1 and BxPC-3 cells	Upregulation	Oncogene	↑ Invasion ↑ Proliferation ↑ Migration	ANKRD1/ANGPTL4	[56]

“↑”: Promotion; “↓”: Suppression.

### 2.2. Colorectal Carcinoma (CRC)

Colorectal carcinoma is widely diagnosed in the digestive system [57]. Functionally, a large number of experiments have proved the biological roles of *AGAP2-AS1* in colorectal carcinoma, including promoting cell growth, migration, invasion and EMT process. Wang et al. circumstantiated that *AGAP2-AS1* promoted colorectal cancer cell growth through modulating the Ras/MAPK pathway. In DLD-1 and HT29 cells, downregulation of *AGAP2*-*AS1* resulted in marked cancer cell growth suppression, cell cycle arrest (at G0/G1 phase) and protein level reduction (p-MEK, p-Raf-1, p-Erk and Ras) [35]. In LoVo and SW480 cells, *AGAP2-AS1* could be transcriptionally activated by E2F4. *AGAP2-AS1* suppression significantly restrained cell growth, migration, invasion and EMT process. Mechanistic studies revealed that *AGAP2-AS1* upregulated CFL1 through competitively combining with miR-182-5p. Meanwhile, restoration of CFL1 counteracted the depleted effects of *AGAP2-AS1* on cancer progression [20]. Similar results were also observed in SW620 and HT-29 cells. Suppression of *AGAP2-AS1* remarkably restrained cancer progression. Mechanistically, *AGAP2-AS1* sponged miR-4668-3p to release SRSF1. Downregulation of miR-4668-3p promoted the malignant processes in *AGAP2-AS1*-knockdown CRC cells, whereas those effects were cancelled out by suppression of SRSF1 [36]. In DLD-1 and RKO cells, Hong et al. demonstrated that *AGAP2-AS1* advanced CRC cancer growth and metastasis, restrained apoptosis and promoted chemoresistance to gemcitabine. The results from mechanistic investigation revealed that *AGAP2-AS1* modulated FGFR1 expression through sponging miR-497 [33]. Furthermore, Ghasemi et al. suggested that *AGAP2-AS1* facilitated CRC progression via sponging the family of hsa-miR-15/16 and upregulating their targets [34]. The above data suggested that *AGAP2-AS1* promoted cell growth, migration, invasion and EMT process of CRC via regulating E2F4/miR-182-5p/CFL1, miR-497/FGFR1, hsa-miR-15/16, Ras/MAPK and miR-4668-3p/SRSF1 pathways.

### 2.3. Lung Carcinoma (LC)

The incidence of LC is over 1.8 million every year, and the death rate is quite high [1,58]. Numerous experiments have confirmed the biological roles of *AGAP2-AS1* in LC, including promotion of cancer growth, metastasis and drug resistance [30,31,32]. In H1299 and H1975 cells, silencing of *AGAP2-AS1* significantly restrained the differentiation and metastasis of cancer cells. Meanwhile, the number of apoptotic cells were obviously increasing. Importantly, the role of *AGAP2-AS1* on promoting tumorigenesis was also verified in in vivo experiments. Mechanistic studies demonstrated that *AGAP2-AS1* bound with EZH2 and LSD1, and recruited them to the promoter regions of KLF2 and LATS2, thereby suppressing the transcription [32]. On the contrary, overexpression of *AGAP2-AS1* prominently activated the differentiation and metastasis of MRC-5 cells. EIF4A3 promoted its stability via binding with *AGAP2-AS1*, which could positively modulate MyD88/NF-κB signaling. Consistently, similar results were also observed in in vivo models [31]. Moreover, the effects of *AGAP2-AS1* on the progression and radio resistance of lung carcinoma were examined. In H157R24-1 and A549R26-1 cells, molecular studies revealed that miR-296 was negatively regulated by *AGAP2-AS1*, while NOTCH2 was a downstream target of miR-296. Mechanistically, the exosomal *AGAP2-AS1* derived by M2 macrophage could enhance the radiotherapy immunity via suppressing miR-296 and activating NOTCH2 [30]. These studies revealed that *AGAP2-AS1* affected the malignant behaviors (promotion of cancer growth, metastasis and drug resistance) of lung cancer via modulating the miR-296/NOTCH2, EIF4A3/MyD88/NF-κB and LATS2/KLF2/EZH2/LSD1 pathways.

### 2.4. Ovarian Carcinoma (OC)

OC, a lethal malignancy in clinic, is commonly occurring in women [59]. The biological functions of *AGAP2-AS1* in OC were investigated. In OVCAR3 and A2780 cell lines, overexpression of *AGAP2-AS1* resulted in significant suppression of MEG3 (*p* < 0.05). Correspondingly, aberrant expression of MEG3 mitigated the function of *AGAP2-AS1* on cell growth. Notably, overexpressed *AGAP2-AS1* did not show significant effects on cell invasion and migration [15]. However, Zheng et al. verified that *AGAP2-AS1* could suppress cell differentiation, migration and invasion in EOC cells. The molecular mechanisms were related to suppression of EMT and downregulation of CTSK, FGFR4 and KRAS [16]. Therefore, the above studies suggested that *AGAP2-AS1* exhibited an important function on cancer proliferation in OC via suppressing MEG3. However, the exact biological functions of *AGAP2-AS1* in EOC cells still needed to be further examined.

### 2.5. Prostate Cancer (PCa)

PCa, mainly observed in males worldwide, is one of the leading causes of cancer death [60]. An increasing number of results have proved the momentous characters of lncRNAs in the tumorigenesis of PCa. Notably, *AGAP2-AS1* was verified as an oncogene in PCa [41,42]. *AGAP2-AS1* was abnormally overexpressed in PCa. Silencing of *AGAP2-AS1* significantly restrained cancer proliferation, migration and invasion. Mechanistically, *AGAP2-AS1* bound with miR-195-5p, which further downregulated the expression of PDLIM5 to obstruct cancer progression. Accordantly, the above mechanisms were also proved in in vivo models [41]. Moreover, Zhao et al. also demonstrated the biological actions of *AGAP2-AS1* in PCa, including restraining apoptotic cell death, promoting proliferation, migration, invasion and EMT process. *AGAP2-AS1* sponged miR-628-5p, which could negatively regulate FOXP2 to affect the cancer growth. Upregulation of FOXP2 reversed *AGAP2-AS1* knockdown-induced suppression on cancer growth [42]. Furthermore, mechanistic studies also enucleated that *AGAP2-AS1* could activate the WNT pathway. Their results suggested that the feedback loop of *AGAP2-AS1*/miR-628-5p/FOXP2 promoted carcinoma growth through activating the WNT signaling [42]. Collectively, *AGAP2-AS1* promoted the proliferation, migration and invasion of PCa through regulating the miR-195-5p/PDLIM5 and miR-628-5p/FOXP2 axes.

### 2.6. Breast Carcinoma (BC)

BC is widely diagnosed in females. It is known as the main reason of carcinomatous fatality [61,62]. The physiological effects of *AGAP2-AS1* in BC were already documented in many studies. Experiments verified that *AGAP2-AS1* was significantly overexpressed in various BC cell lines and clinical samples [43]. Silencing of *AGAP2-AS1* remarkably restrained cell proliferation and increased apoptotic cell death in MCF-7 (ER+) cells. Mechanistical results revealed that *AGAP2-AS1* bound with HuR to upregulate H3K27ac, thereby elevating the activity of MTA1 promoter and upregulating MTA1 expression. High expression of H3K27ac partially offset the function of si-*AGAP2-AS1*-mediated apoptosis induction. This mechanism was also verified in in vivo models [43]. In addition, the aberrant expression of *AGAP2-AS1* was also detected in drug-resistant cells. In trastuzumab-resistant cells (HER2-positive BT474 and SKBR-3 cells), *AGAP2-AS1* knockdown promoted the inhibitory effects of trastuzumab on cell growth. Experimental data indicated that the excreted *AGAP2-AS1* was assembled into exosomes in an hnRNPA2B1-reliant way. Co-treatment exosomes with trastuzumab remarkably decreased the inhibitory effects of trastuzumab on cell growth [12]. Hence, *AGAP2-AS1* could promote the progression (proliferation and drug resistance) of BC via regulation of HuR/H3K27ac/MTA1 and hnRNPA2B1 pathways.

### 2.7. Cholangiocarcinoma (CHOL)

The biological characters of *AGAP2-AS1* in CHOL were also assessed. Ji et al. demonstrated that *AGAP2-AS1* could promote the CHOL cell growth. *AGAP2-AS1* influenced CDKN1A transcription by interacting with EZH2 in cancer cells. Their results illustrated that *AGAP2-AS1* suppression significantly restrained the growth of HUCCT1 and RBE cells. Importantly, SP1 could positively regulate *AGAP2-AS1* expression. Suppression of *AGAP2-AS1* led to a remarkable increase in apoptotic cells [44]. Moreover, in RBE and HuCCT-1 cells, experiments illustrated that inhibition of GOLGA7B significantly facilitated cell migration and invasion, while knockdown of *AGAP2-AS1* exhibited the reverse effects [45]. The above results suggested that *AGAP2-AS1* facilitated cell growth, migration and invasion by modulating CDKN1A and GOLGA7B pathways.

### 2.8. Bladder Cancer (BLCA)

The characters of *AGAP2-AS1* in BLCA were also reported in some studies. In UM-UC-3 and T24 cells, Xu et al. demonstrated that silencing of *AGAP2-AS1* could obviously restrain cell proliferation and migration [46]. In contrast, overexpression of *AGAP2-AS1* significantly facilitated cancer progression in in vitro and in vivo model systems, including promotion of proliferation, invasion, migration and angiogenesis. Suppression of *AGAP2-AS1* displayed reverse effects. Mechanistic studies demonstrated that *AGAP2-AS1* could directly bind to IGF2BP2 to promote LRG1 stability. Meanwhile, overexpression of LRG1 could reverse the *AGAP2-AS1*-knockdown mediated effects in cancer cells [47]. These data proved that *AGAP2-AS1* advanced cancer proliferation, invasion, migration and angiogenesis through regulating the IGF2BP2/LRG1 axis.

### 2.9. Colon Cancer (CLC)

Colon cancer is commonly diagnosed in the digestive system. The incidence is second only to lung and liver cancer [1,63]. Ji et al. documented that *AGAP2-AS1* and LINC-PINT could form a negative feedback loop to promote CLC progression. Their data indicated that *AGAP2-AS1* suppressed LINC-PINT in RKO and HCT116 cells. Meanwhile, suppression of LINC-PINT promoted the expression of *AGAP2-AS1*. Experiments also proved that *AGAP2-AS1* could significantly promote cancer progression via regulating Hippo signaling [37]. In SW480 and HCT-116 cells, highly expressed *AGAP2-AS1* was also verified [38,39,40]. Inhibition of *AGAP2-AS1* led to a reduction in cell proliferation and migration [38,39,40]. Notably, *AGAP2-AS1* knockdown triggered the phosphorylation of YAP and led to a marked decrease in MMP-9 and MMP-2. Further results proved that *AGAP2-AS1* upregulated MMPs via activation of the YAP pathway (*p* < 0.05) [40]. However, Liu et al. indicated that *AGAP2-AS1* displayed the biological functions via targeting miR-646 in HCT-116 cells [39]. In summary, *AGAP2-AS1* promoted cancer proliferation, migration and invasion of CLC cells via regulating LINC-PINT/Hippo, miR-646 and YAP pathways.

### 2.10. Pancreatic Cancer (PC)

The characters of *AGAP2-AS1* in PC were also assessed. In AsPC-1 and BxPC-3 cells, *AGAP2-AS1* significantly affected cancer progression in vitro and in vivo. It was documented that RREB1 could positively regulate the *AGAP2-AS1* transcription. Studies indicated that *AGAP2-AS1* epigenetically stifled ANKRD1 and ANGPTL4 through recruiting EZH2, thereby facilitating cancer proliferation and metastasis [56]. These data demonstrated that *AGAP2-AS1* facilitated PC proliferation and metastasis through the RREB1/ANKRD1/ANGPTL4 pathway.

### 2.11. Renal Cell Carcinoma (RCC)

*AGAP2-AS1* was demonstrated to be highly overexpressed in RCC. In 786-O and ACHN cells, experiments verified that *AGAP2-AS1* could be stabilized by IGF2BP3 via m6A modification. Overexpression of *AGAP2-AS1* promoted pernicious behaviors of RCC cells and resulted in M2 polarization. Functionally, *AGAP2-AS1* directly sponged miR-9-5p to upregulate the expression of THBS2. On the other side, THBS2 could subsequently improve macrophage polarization via regulating the PI3K/AKT pathway [48]. Taken together, *AGAP2-AS1* promoted the proliferation and tumor growth of RCC through regulation of the miR-9-5p/THBS2/PI3K-AKT axis.

### 2.12. Laryngeal Squamous Cell Carcinoma (LSCC)

LSCC is a highly malignant and invasive carcinoma. Experiments demonstrated that *AGAP2-AS1* was markedly overexpressed in Tu-177 and AMC-HN-8 cell lines. Suppression of *AGAP2-AS1* obviously restrained cell growth and invasion. Mechanistically, *AGAP2-AS1* could sponge miR-193a-3p to regulate its expression. In addition, LOXL4 was demonstrated as a direct target of miR-193a-3p. In brief, those results illustrated that *AGAP2-AS1* regulated the miR-193a-3p/LOXL4 axis to facilitate cancer growth and invasion [55].

### 2.13. Melanoma

The biological characters of *AGAP2-AS1* in melanoma were also reported. Results indicated that *AGAP2-AS1* was overexpressed in SKCM tissues. Its expression was associated with poor prognosis. Silencing of *AGAP2-AS1* in A875 and A375 cells evidently suppressed cell growth and migration in various model systems. Experimental data demonstrated that *AGAP2-AS1* could interact with BRD7. Silencing of *AGAP2-AS1* alleviated the interaction of BRD7 and c-Myc, which could further decrease the expression of c-Myc. Notably, c-Myc overexpression reversed the biological effects in *AGAP2-AS1*- and BRD7-deficient cells [54]. These results demonstrated that *AGAP2-AS1* is involved in oncogenesis (cell growth and migration) through regulating the BRD7/c-Myc pathway.

### 2.14. Papillary Thyroid Cancer (PTC)

*AGAP2-AS1* also showed significant functions on promotion of cell growth, migration and invasion in PTC cells [49,50]. Restraint of *AGAP2-AS1* prominently alleviated the migration and invasion of K1 and TPC1 cells. Mechanistic studies revealed that *AGAP2-AS1* could competitively bind to miR-425-5p to upregulate the level of MMP-2, thereby promoting cell migration and invasion. Moreover, clinical studies disclosed that miR-424-5p was evidently downregulated in cancerous samples. It was negatively correlated with the expression of *AGAP2-AS1* [49]. In addition, Xu et al. verified that *AGAP2-AS1* exerted the biological effects via regulation of the miR-628-5p/KLF12 axis. Their results proved that the level of *AGAP2-AS1* was negatively correlated with miR-628-5p. Mechanistically, *AGAP2-AS1* sponged miR-628-5p to directly regulate KLF12 expression. Importantly, silencing of KLF12 increased the inhibitory functions of miR-628-5p on cancer growth and metastasis [50]. Collectively, *AGAP2-AS1* promoted the proliferation, migration and invasion of PTC via regulating miR-425-5p/MMP-2 and miR-628-5p/KLF12 axes.

### 2.15. Esophageal Cancer (EC)

The effects of *AGAP2-AS1* on EC were also examined. Experimental results proved that *AGAP2-AS1* suppression or miR-195-5p overexpression could strikingly arrest cell cycle, promote apoptotic cell death, and alleviate proliferation, migration and invasion. Functionally, *AGAP2-AS1* could bind to miR-195-5p to regulate FOSL1. Conversely, *AGAP2-AS1* knockdown preeminently suppressed the cancer progression via upregulation of miR-195-5p and downregulation of FOSL1 [53]. Moreover, it was well known that the antisense lncRNAs were usually acting as regulators of their sense counterparts. Consistently, Zheng et al. (2020) proved that the expression of *AGAP2-AS1* in EOC tissues was negatively correlated with that of AGAP2 [61].

### 2.16. Gastric Cancer (GC)

The biological roles of *AGAP2-AS1* in GC were verified in various studies [51,52]. Functional studies revealed that silencing of *AGAP2-AS1* led to suppression of cell proliferation and migration [51,52]. Data from mechanistic experiments uncovered that *AGAP2-AS1* bound with WTAP to facilitate the WTAP/METTL3/METTL14 complex. Meanwhile, STAT3 mRNA was stabilized by *AGAP2-AS1* in an m6A-reliant way, thereby activating the IL-6/STAT3 pathway. Notably, *AGAP2-AS1*/WTAP/STAT3 pathway activation memorably facilitated cell proliferation and migration [51]. Moreover, Qi et al. demonstrated that SP1 could upregulate *AGAP2-AS1* expression in AGS and BGC-823 cells. Functionally, *AGAP2-AS1* interacted with LSD1 and EZH2 to inhibit the transcription of p21 and E-cadherin, resultingly exerting oncogenic functions [52]. In summary, *AGAP2-AS1* affected the proliferation and migration of GC by regulating the WTAP/STAT3/METTL3/METTL14 and LSD1/EZH2/p21/E-cadherin pathways.

## 3. Clinical Significances

**Figure 3:**
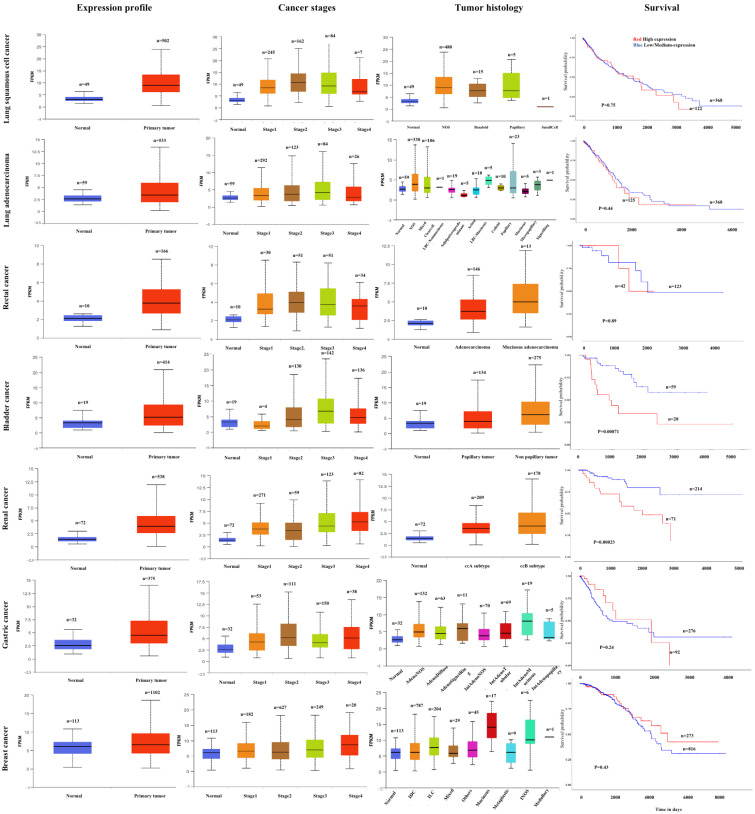
The *AGAP2-AS1* expression profile, and its correlation with cancer stage, tumor histology and survival analyzed using the UALCAN database (https://ualcan.path.uab.edu/, accessed on 20 May 2024).

**Figure 4:**
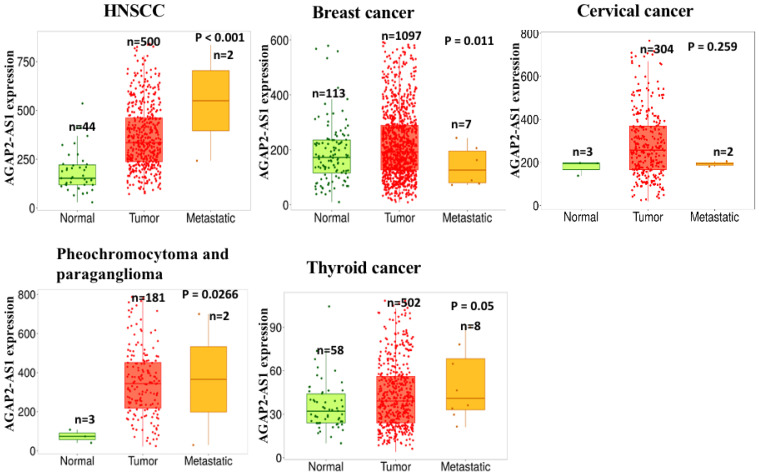
The *AGAP2-AS1* expression in normal, tumor, and metastatic tumor tissues using the TNM plot (https://tnmplot.com/analysis/, accessed on 22 May 2024).

The expression level of *AGAP2-AS1* in clinical carcinomatous and non-carcinomatous tissues was analyzed using the UALCAN database (https://ualcan.path.uab.edu/, accessed on 18 May 2024) [64,65]. As shown in Figure 1, *AGAP2-AS1* was significantly overexpressed in a large number of carcinomas, including glioma, LC, CLC, OC, PTC, CHOL, BLCA, CRC, PC and ccRCC. Experimental analysis indicated that the expression of *AGAP2-AS1* in some tumors was closely correlated with TS, histology, survival and cancer metastasis (Figure 3). Meanwhile, the data from the TNM plot (https://tnmplot.com/analysis/, accessed on 18 May 2024) also proved the high expression of *AGAP2-AS1* in tumor and metastatic tissues (Figure 4) [66]. The clinical significances of *AGAP2-AS1* in human cancers are indicated in Table 2.

### 3.1. Gliomas

The clinical significances of *AGAP2-AS1* in gliomas were widely documented by dataset analysis. Wang et al. divided anaplastic gliomas into grade II- and grade IV-like groups using datasets. Multivariate analysis indicated that *AGAP2-AS1* was evidently raised with TG [23]. Correspondingly, similar results were demonstrated by Wang et al. [67]. Luo et al. constructed a ceRNA network of *AGAP2-AS1*-miR-9-5p-MMP-2/MMP-9 and identified that *AGAP2-AS1* was a promising therapeutic target for GBM [27]. In addition, *AGAP2-AS1* was also identified as one of the immune-related lncRNAs in patients with gliomas [29,67]. The signature of *AGAP2-AS1* exhibited prognostic values for anaplastic gliomas [67]. Interestingly, *AGAP2-AS1* was not only an independent prognostic factor but also could effectively predict the survival rate of patients [68]. However, Yu et al. verified that GBM subtype-A was featured by low expression of *AGAP2-AS1* [29].

On the other hand, clinical samples were also applied to assess the clinical relevance of *AGAP2-AS1*. Abnormal overexpression of *AGAP2-AS1* was observed in several studies [19,25,26]. Tian et al. documented that the expression of *AGAP2-AS1* was significantly overexpressed in 136 glioma tissue specimens. Kaplan–Meier analysis revealed that the *AGAP2-AS1* expression was negatively associated with OS in GBM patients [19]. In a cohort of 91 pairs of glioma tissues, the level of *AGAP2-AS1* in carcinomatous samples was higher than that in non-carcinomatous samples. Noticeably, the aberrant expression of *AGAP2-AS1* was highly correlated with advanced TG and low OS rate [26]. In addition, Luo et al. also obtained the similar data from 58 paired tissues. GEPIA data analysis disclosed that *AGAP2-AS1* overexpression in patients indicated a shorter OS [25]. Collectively, the above studies demonstrated that the expression of *AGAP2-AS1* in clinical glioma tissues was highly associated with advanced TG and OS.

### 3.2. Lung Carcinoma

The abnormal level of *AGAP2-AS1* in lung carcinoma was widely verified. In a cohort of 121 pairs of lung carcinoma samples (84 radioresistant and 37 radioresistant cases), *AGAP2-AS1* was observably upregulated in carcinomatous samples. Compared with the radiosensitive groups, radioresistant patients showed a much higher level of *AGAP2-AS1*. Importantly, the *AGAP2-AS1* level was closely related to the TNM stage and LNM. However, other factors, such as tumor differentiation, age and pathological pattern, did not exhibit any correlation with the expression of *AGAP2-AS1*. Moreover, Kaplan–Meier analysis indicated that the high level of *AGAP2-AS1* in patients was tightly linked to lower OS and DFS [30,32,69]. Meanwhile, the abnormal expression of *AGAP2-AS1* in NSCLC patients was also extensively demonstrated [32,70,71,72,73,74,75]. Experiments revealed that the level of *AGAP2-AS1* was highly correlated with TNM stage, LNM and poor prognostic outcomes [32,72,73,74,75]. In another study with 84 patients, the expression of serum *AGAP2-AS1* in NSCLC patients was associated with differentiation degree [75]. In a cohort of 80 pairs of NSCLC tissues, the *AGAP2-AS1* expression increased 41.5-fold in 72.5% of cancerous tissues. Importantly, increased *AGAP2-AS1* level was also correlated with tumor size in NSCLC patients [32]. Ma et al. demonstrated that the survival rate of patients with low expression of *AGAP2-AS1* was 63.64% (28/44), which was significantly higher than 34. 21% (13/38) of patients with high expression of *AGAP2-AS1* [72]. Moreover, in a cohort of 198 pairs of NSCLC tissues, results indicated that the *AGAP2-AS1* level in NSCLC was closely linked with tumor stage (TS) and LNM. Kaplan–Meier analysis illustrated that patients with high expression of *AGAP2-AS1* showed a shorter OS time (*p* < 0.001). Meanwhile, multivariate analysis demonstrated that *AGAP2-AS1* was an independent prognostic factor of OS [73]. In addition, Zhang et al. verified that the aberrant level of *AGAP2-AS1* in NSCLC was closely associated with clinical stage, tumor infiltration and LNM [74]. In summary, the above data proved that the expression of *AGAP2-AS1* in LC tissues was highly correlated with TNM stage, LNM, lower OS, DFS, poor prognostic outcome, tumor size, tumor infiltration and differentiation degree.

**Table 2:** Clinical significance of *AGAP2-AS1* in human carcinomas.

Cancer	Property	Samples	Clinic-Pathological Features	Ref.
Gliomas	Oncogene	GEO: 9 paired tissues TCGA: 169 GBM cases and 5 normal samples	Correlated with occurrence and development of glioblastoma.	[27]
	Oncogene	GSE16011, CGGA and REMBRANDT datasets	Correlated with TG.	[23]
	Oncogene	CGGA dataset: 51 paired tissues	Correlated with TG.	[67]
	Oncogene	136 cancer tissues and 20 normal tissues	Negatively correlated with OS.	[19]
	Oncogene	91 paired tissues	Associated with the advanced TG.	[26]
	Oncogene	58 paired tissues	Correlated with OS.	[25]
LC	Oncogene	121 LC patients	Related to the TNM stage and LNM.	[30]
	Oncogene	198 paired tissues	Correlated with TG and LNM.	[73]
	Oncogene	150 patients with NSCLC, including 86 ADCs and 64 SCCs 150 healthy controls	Correlated with LNM and TNM stage.	[71]
	Oncogene	80 NSCLC patients	Served as a potential independent prognostic value in NSCLC.	[32]
	Oncogene	120 patients with NSCLC 60 healthy controls	Correlated with clinical stage, tumor infiltration and LNM.	[74]
	Oncogene	82 paired tissues	Correlated with occurrence and development of NSCLC.	[72]
	Oncogene	84 NSCLC patients 60 healthy controls	Correlated with LNM, TNM stage and differentiation degree.	[75]
	Oncogene	535 tumor samples and 59 normal samples	Correlated with total patient survival.	[69]
	Oncogene	14 smokers, 17 NSCLC patients and 14 healthy subjects	Correlated with the development of NSCLC.	[70]
OC	Oncogene	82 paired tissues	Correlated with clinical stage and the expression levels of MEG3.	[15]
	Anti-oncogene	80 cancerous tissues and 10 normal tissues	Associated with advanced FIGO stage, high HG, LNM and serous subtype.	[16]
PTC	Oncogene	110 paired tissues	Associated with invasion and migration.	[49]
	Oncogene	31 paired tissues	Not stated.	[50]
CHOL	Oncogene	TCGA and GEO databases (not stated)	Correlated with metabolism-related mechanisms in tumorigenesis.	[76]
	Oncogene	TCGA: 36 cancer tissues and 9 normal tissues	Correlated with survival time.	[44]
	Oncogene	9 paired tissues	Correlated with survival time.	[45]
BLCA	Oncogene	33 paired tissues	Associated with higher possibility of recurrence.	[46]
	Oncogene	45 paired tissues	Positively correlated with T stage, grade and vascular invasion; negatively correlated with the survival.	[47]
RC	Oncogene	50 paired tissues	Abnormally overexpressed.	[35]
	Oncogene	70 paired tissues	Abnormally overexpressed.	[20]
	Oncogene	116 paired tissues	Highly correlated to TG.	[33]
	Oncogene	100 paired tissues	No association with the clinicopathological characteristics.	[34]
CLC	Oncogene	TCGA: 457 cancer samples and 42 healthy samples 20 paired tissues	Aberrantly overexpressed.	[40]
	Oncogene	66 paired tissues	Not significantly correlated with TG.	[37]
ccRCC	Oncogene	539 ccRCC tissues and 72 adjacent healthy tissues	Associated with poor OS.	[77]
	Oncogene	443 paired tissues	Correlated with overall unfavorable survival outcome.	[18]
	Oncogene	50 paired tissues	Associated with poor survival and prognosis.	[48]
Melanoma	Oncogene	468 cancer tissues and 555 corresponding normal tissues	Associated with shorter OS, disease-specific survival and PFI times.	[54]
GC	Oncogene	GSE13911: 69 paired tissues GSE54129: 27 paired tissues	Associated with adverse OS and PFS in advanced-stage (III–IV) GC.	[51]
	Oncogene	50 paired tissues	Associated with larger tumors, advanced pathological stage and LNM.	[52]
PC	Oncogene	16 paired tissues 46 paired tissues	Associated with poor prognosis.	[56]
LSCC	Oncogene	23 paired tissues	Associated with the metastasis of LSCC.	[55]
BC	Oncogene	78 paired tissues	Associated with differentiate malignant states.	[78]
	Oncogene	30 paired tissues	Aberrantly overexpressed.	[43]
PCa	Oncogene	50 cancerous tissues and 20 benign tissues	Aberrantly overexpressed.	[41]

### 3.3. OC

OC is a conventional carcinoma in females. In a cohort of 82 paired OC tissues, RT-qPCR analysis indicated that *AGAP2-AS1* noticeably increased in cancerous tissues compared with the adjacent non-cancerous tissues. Importantly, the aberrant expression of *AGAP2-AS1* was highly correlated with advanced clinical stages [15]. Moreover, studies also revealed that MEG3 was downregulated in tumor tissues. Linear regression analysis demonstrated that the levels of *AGAP2-AS1* and MEG3 were negatively associated in tumor samples [15]. In another study, Zheng et al. illustrated that *AGAP2-AS1* remarkably decreased in 267 OC tissues in the GEO database. Their further results verified that *AGAP2-AS1* was observably reduced in 80 EOC samples. Importantly, the low expression of *AGAP2-AS1* was tightly connected with advanced high HG, serous subtype, FIGO stage and LNM [16]. These data indicated that the expression of *AGAP2-AS1* in OC was significantly related to clinical stage, high HG, serous subtype, FIGO stage and LNM.

### 3.4. PTC

In a cohort of 31 paired PTC tumor samples, the *AGAP2-AS1* level was assessed by RT-qPCR. Results indicated that *AGAP2-AS1* was noticeably upregulated in cancerous tissues [50]. In addition, the level of *AGAP2-AS1* in 110 paired PTC tissues was also analyzed. Compared with non-cancerous tissues, the expression of *AGAP2-AS1* in cancer tissues was much higher than that in non-cancerous tissues. Importantly, the level of *AGAP2-AS1* was significantly correlated with the TNM stage (*p* < 0.01) and LNM (*p* < 0.05) [49]. Therefore, those results revealed that the *AGAP2-AS1* level in PTC showed obvious correlation with TNM stage and LNM.

### 3.5. CHOL

The *AGAP2-AS1* level in CHOL tissues was also assessed. In a cohort of 32 pairs of CHOL tissues, experimental results indicated that *AGAP2-AS1* was markedly overexpressed in tumors [44]. Ji et al. examined the expression of *AGAP2-AS1* in 36 cancerous tissues and 9 non-cancerous tissues using the TCGA database. The aberrant expression of *AGAP2-AS1* was confirmed. In accordance with the survival analysis, experimental results proved that the elevated *AGAP2-AS1* level was associated with short survival time [44]. Similar results were also observed in another study [45]. Hence, *AGAP2-AS1* could be applied as an important prognostic signature to independently predict prognosis [76].

### 3.6. BLCA

The high expression of *AGAP2-AS1* was also demonstrated in patients with BLCA [46,47]. The survival analysis of *AGAP2-AS1* in 33 BLCA samples illustrated that the overexpression of *AGAP2-AS1* was closely associated with the probability of BLCA recrudescency (*p* < 0.05) [46]. In a cohort of 45 paired BLCA tissues, *AGAP2-AS1* was significantly overexpressed in tumors. In addition, the *AGAP2-AS1* level in stages III and IV was much higher than that in stage II. Importantly, the expression of *AGAP2-AS1* was negatively associated with the patient survival, but positively associated with vascular invasion, grade and T stage [47].

### 3.7. Rectal Cancer (RC)

The aberrant overexpression of *AGAP2-AS1* was markedly observed in RC tissues in several studies [20,35]. In a study of 116 cases of RC, experiments documented that the *AGAP2-AS1* expression was negatively correlated with the patient survival. *AGAP2-AS1* was noticeably overexpressed in 48.3% (56 of 116) RC tissues. Furthermore, the expression of *AGAP2-AS1* was tightly linked to TS. Survival analysis disclosed that the OS and DFS in the overexpressed group were noticeably shorter than that in the low group [33]. Meanwhile, Ghasemi et al. proved that the *AGAP2-AS1* level was 4-fold higher in tumor samples than that in the non-tumor samples. However, there was no correlation between the clinical features and the *AGAP2-AS1* level [34].

### 3.8. PC

In a cohort of 46 pairs of PC tissues, RT-qPCR was applied to analyze the *AGAP2-AS1* level in tissues. Experiments indicated that *AGAP2-AS1* obviously increased in 78.3% (36/46) PC tissues. The level of *AGAP2-AS1* in tumors was remarkably connected with LNM, late TNM staging and tumor size. Whereas, *AGAP2-AS1* showed no evident correlation with age, HG and gender. Notably, Kaplan–Meier analysis revealed that aberrant expression of *AGAP2-AS1* in PC patients usually represented poor prognosis (*p* < 0.01) [56].

### 3.9. CLC

The TCGA analysis from 457 CLC samples and 42 healthy samples indicated that the *AGAP2-AS1* level was evidently overexpressed in colon tumor tissues. Meanwhile, Jin et al. examined the level of *AGAP2-AS1* in 20 matched colon carcinoma and adjacent tissues. Consistently, *AGAP2-AS1* remarkably increased in cancer tissues [40]. Homologous results were demonstrated in a study with 66 cases. However, Ji et al. verified that the *AGAP2-AS1* level in cancer tissues was not evidently correlated with TS [37].

### 3.10. ccRCC

In a cohort of 443 ccRCC patients, experimental results verified the overexpression of *AGAP2-AS1* in cancer samples. Meanwhile, the level of *AGAP2-AS1* was closely associated with unfavorable survival outcomes [18]. Gao et al. detected the *AGAP2-AS1* level in 539 tumor tissues and 72 adjacent non-tumor tissues. Their data illustrated that the upregulated *AGAP2-AS1* in tumor tissues was observably correlated with distant metastasis, LNM, T3/T4, worse prognosis, male, poor OS and high TG (III/IV) [77]. Moreover, in a cohort of 50 pairs of ccRCC tissues, authors illustrated that the abnormal level of *AGAP2-AS1* was highly connected with poor survival and prognosis in RCC patients [48]. Furthermore, Zhang et al. found that the prognostic signature containing *AGAP2-AS1* was established as a promising biomarker for ccRCC prognosis [79].

### 3.11. Melanoma

The clinical significances of *AGAP2-AS1* in melanoma were analyzed by the TCGA database. Results demonstrated that *AGAP2-AS1* in SKCM tissues prominently increased. Highly expressed *AGAP2-AS1* was notably correlated with advanced stage in SKCM. Importantly, SKCM patients with overexpressed *AGAP2-AS1* were commonly accompanied with DSS, shorter OS and PFI times [54].

### 3.12. GC

Nie et al. analyzed the datasets of GSE13911 (*n* = 69) and GSE54129 (*n* = 27). Their results documented that patients with higher *AGAP2-AS1* level had shorter OS, PFS and PPS. The Kaplan–Meier plotter indicated that overexpression of *AGAP2-AS1* was related to adverse OS and PFS in advanced-stage (III-IV) GC, as well as HER2-negative and -positive status [51]. In another study, 50 paired GC tissues were collected. *AGAP2-AS1* was obviously overexpressed in cancer tissues. Importantly, high *AGAP2-AS1* level indicated a poor prognosis, shorter OS, large tumors, advanced TS and LNM [52].

### 3.13. BC

Wu et al. assessed the level of *AGAP2-AS1* in 30 paired BC tissues. Experimental data demonstrated the high *AGAP2-AS1* level in tumor tissues (*p* < 0.05) [43]. However, Mohebi et al. proved that *AGAP2-AS1* in 78 tumoral tissues was evidently lower than that in non-cancerous tissues. They also proved that the expression level of *AGAP2-AS1* was lower in patients with menarche age between 10 and 12 years old [78]. In addition, Hisey et al. investigated the RNA expression profiles in 15,741 tumor and non-tumor samples in the TCGA and GTEx databases. Whereas, there were no significant differences of *AGAP2-AS1* between tumor and nontumor tissues [80]. Therefore, they concluded that *AGAP2-AS1* was not a potential biomarker in breast cancer [80].

### 3.14. Other Cancers

The investigation of *AGAP2-AS1* in other cancers was also reported. In a cohort of 23 paired LSCC tissues, the level of *AGAP2-AS1* was markedly higher in LSCC tissues than that in non-cancerous tissues by RT-qPCR analysis [55]. In another study, RT-qPCR also demonstrated a remarkable increase in *AGAP2-AS1* in prostate cancerous samples (*p* < 0.05) [41]. Nevertheless, their clinical significances of *AGAP2-AS1* in LSCC and prostate cancer were not fully illustrated.

## 4. Future Perspectives

To date, a great number of experiments have demonstrated that *AGAP2-AS1* plays an important character in cancer progression, such as cancer proliferation, migration, invasion and EMT process. Notably, *AGAP2-AS1* was proven as a new oncogene in human cancers. Experimental data proved that *AGAP2-AS1* was involved in promoting cancer proliferation, migration and invasion. Importantly, *AGAP2-AS1* also showed obvious clinical significances. High level of *AGAP2-AS1* was closely associated with poor survival in patients, indicating its potential as a prognostic marker. Clinical results showed that aberrant overexpression of *AGAP2-AS1* was significantly connected with OS, LNM, clinical stage, tumor infiltration, high HG, serous subtype and PFI times.

Since *AGAP2-AS1* is frequently overexpressed in various human cancers, targeting AGAP2-AS1 could probably achieve therapeutic purposes in the future. The strategies of targeting *AGAP2-AS1* are diverse. Currently, compounds such as DAPT and berberine could significantly suppress cancer progression via targeting lncRNAs [81,82]. Therefore, development of small molecules from chemicals could be a potential way to inhibit *AGAP2-AS1* in cancers. Secondly, small interfering RNAs (siRNAs) complementary to *AGAP2-AS1* could be applied for targeted therapy. siRNAs could remarkedly decrease the expression of targeted lncRNAs, thereby suppressing the proliferation, migration and invasion of cancer cells [83]. Moreover, *AGAP2-AS1* was demonstrated as an immune-related lncRNA in patients with gliomas [29,67]. Hence, the approach of immune response targeting could be another way to regulate *AGAP2-AS1*. Finally, the CRISPR genome editing and targeting of upstream molecules of *AGAP2-AS1* are also promising approaches.

Even though the biological activities and clinical features of *AGAP2-AS1* have been extensively proved in many cancers, much work is still required to explore the biological role of *AGAP2-AS1* in various cancers. Future studies should focus on elucidating the possible molecular mechanisms, studying epigenetic modifications and exploiting *AGAP2-AS1*-related manners for cancer diagnosis and treatment. Mechanistically, the molecular mechanisms and clinical significances of *AGAP2-AS1* in many carcinomas are still not completely clarified. The upstream regulators and downstream targets of *AGAP2-AS1* in many cancers are still unclear. Furthermore, studies regarding the role of *AGAP2-AS1* in immune response and tumor microenvironment are still limited. Finally, it was reported that lncRNAs could synchronously regulate numerous targets, thereby triggering a wide range of unconversant physiologic and pathologic responses. To avert the possible side effects resulted from off-targeting, it is momentous to clarify the pathophysiological mechanism of *AGAP2-AS1* before clinical application.

## 5. Conclusions

Collectively, the present overview concluded the biological activities, molecular mechanisms and clinical features of *AGAP2-AS1* in various carcinomas. *AGAP2-AS1* played a critical role in cancer progression. Meanwhile, the clinical significances of *AGAP2-AS1* were also verified in a number of studies. Hence, *AGAP2-AS1* is a promising biomarker for cancer therapy.

## Data Availability

Not applicable.

## Abbreviations

ANGPTL4: Angiopoietin-like 4; ANKRD1: Ankyrin repeat domain 1; BC: Breast carcinoma; BLCA: Bladder cancer; BRD7: Bromodomain containing 7; CHOL: Cholangiocarcinoma; ccRCC: Clear cell renal cell carcinoma; CDKN1A: cyclin-dependent kinase inhibitor 1A; CFL1: Cofilin-1; CLC: Colon cancer; CRC: Colorectal cancer; CTSK: Cathepsin-K; DFS: Disease-free survival; DSS: Disease-specific survival; E2F4: E2F transcription factor 4; EC: Esophageal cancer; EIF4A3: Eukaryotic initiation factor 4A3; EMT: Epithelial–mesenchymal transition; EOC: Epithelial ovarian cancer; EZH2: Enhancer of zeste homolog 2; FGFR: Fibroblast growth factor receptor; FOXP2: Forkhead box protein 2; Fosl1: Fos-like antigen 1; GC: Gastric cancer; H3K27ac: Histone H3 lysine 27 acetylation; HDGF: Hepatoma-derived growth factor; HG: Histological grade; HNSCC: Head and neck squamous cell carcinoma; HuR: Hu-antigen R; IGF2BP2: Insulin-like growth factor 2 mRNA binding protein 2; KLF: Kruppel-like factor; KRAS: Kirsten rat sarcoma; LATS2: Large tumor suppressor kinase; LC: Lung cancer; LINC-PINT: Long intergenic non-protein-coding RNA p53-induced transcript; LncRNAs: Long non-coding RNAs; LNM: Lymph node metastasis; LOXL4: Lysyl oxidase-like 4; LSCC: Laryngeal squamous cell carcinoma; LSD1: Lysine (K)-specific demethylase 1A; MAPK: Mitogen-activated protein kinases; MEG3: Maternally expressed gene 3; METTL3: Methyltransferase-like 3; MMPs: Matrix metalloproteinases; MTA1: Metastasis-associated 1; MyD88: Myeloid differentiation factor-88; NF-κB: Nuclear factor kappa B; NSCLC: Non-small-cell lung cancer; OC: Ovarian carcinoma; OS: Overall survival; PCa: Prostate cancer; PC: Pancreatic cancer; PDLIM5:PDZ and LIM domain 5 gene; PFS: Progression-free survival; PPS: Post-progression survival; PTC: Papillary thyroid cancer; RC: Rectal cancer; RCC: Renal cell carcinoma; SKCM: Skin cutaneous melanoma; SRSF1: serine/arginine-rich splicing factor 1; TG: Tumor grade; THBS2: Thrombospondin-2; TFPI2: Tissue factor pathway inhibitor 2; TNM: Tumor node metastasis; TS: Tumor stage; WTAP: WT1-associated protein.
